# Optimism may moderate screening mammogram frequency in Medicare: A longitudinal study: Erratum

**DOI:** 10.1097/MD.0000000000016380

**Published:** 2019-07-05

In the article, “Optimism may moderate screening mammogram frequency in Medicare: A longitudinal study”,^[1]^ which appears in Volume 98, Issue 24 of *Medicine*, Dr. Milagros C. Rosal's name was missing the middle initial and has an MS as well as a PhD. Dr. Rosal's affiliation should appear as Department of Population and Quantitative Health Sciences, University of Massachusetts Medical School, Worcester, MA. Dr. Julie Donohue's affiliation should appear as University of Pittsburgh Graduate School of Public Health.
